# Cost-effectiveness analysis of gumarontinib versus savolitinib for the treatment of advanced or metastatic NSCLC with MET exon 14 skipping mutations in China using partitioned survival model

**DOI:** 10.3389/fphar.2025.1400422

**Published:** 2025-01-24

**Authors:** Gang Fang, Zhipeng Pi, Yiping An, Xinxin Cao, Wei Li, Xiangjun Zhu, Jinxi Ding

**Affiliations:** ^1^ School of International Pharmaceutical Business, China Pharmaceutical University, Nanjing, China; ^2^ Pharmaceutical Market Access Policy Research Center, China Pharmaceutical University, Nanjing, China; ^3^ Jiangsu Health Development Research Center, Nanjing, China

**Keywords:** cost-effectiveness analysis, METex14, non-small-cell lung cancer, gumarontinib, savolitinib, MAIC, partitioned survival model, Chinese healthcare system

## Abstract

**Background and objectives:**

Both gumarontinib and savolitinib have demonstrated efficacy in treating non-small-cell lung cancer (NSCLC) with tumors harboring mesenchymal–epithelial transition factor gene exon 14 (METex14) skipping. However, the comparison of their efficacy and pharmacoeconomics profiles remains limited. This study aims to evaluate the cost-effectiveness of gumarontinib versus savolitinib for the treatment of METex14 skipping NSCLC in China.

**Methods:**

A 3-state partitioned survival model (PSM) was developed with lifetime horizon from the perspective of Chinese healthcare system. Survival inputs were based on an unanchored matching-adjusted indirect comparison using individual patient data from GLORY trial to adjust for patient characteristics in NCT02897479. Costs and outcomes were discounted at an annual rate of 5%. Sensitivity and scenario analyses were conducted to explore model uncertainty.

**Results:**

Gumarontinib gained an additional 0.10 QALYs at an incremental cost of $1,893 compared to savolitinib, resulting in the ICERs of $19,243/QALY, which is below the threshold of 3 times the GDP *per capita* in China ($35,007 *per capita* in 2022). Sensitivity and scenario analyses confirmed the robustness of the base-case results.

**Conclusion:**

Gumarontinib is a cost-effective option compared to savolitinib for METex14 skipping NSCLC in China.

## 1 Introduction

Lung cancer is one of the most commonly diagnosed cancers worldwide, with an estimated 2.5 million new cases in 2022, according to the International Agency for Research on Cancer (IARC) (Sung et al., 2021; Leiter et al., 2023). In China, the 2022 report by the National Cancer Center (NCC) indicated 1,060,600 new cases and 733,300 deaths, ranking first among all malignant tumors (Zheng et al., 2022; Han et al., 2024). Furthermore, lung cancer accounted for 17,128,580 disability-adjusted life years (DALYs) in 2019, representing approximately 24.3% of DALYs caused by all cancers, thereby imposing a significant disease burden on society (Sun et al., 2020; Fang et al., 2023).

Lung cancer is classified into small-cell lung cancer (SCLC) and non-small-cell lung cancer (NSCLC), with NSCLC comprising 80%–85% of all lung cancer cases (Duma et al., 2019). Among Chinese patients with NSCLC, 0.9%–2.0% harbor mesenchymal–epithelial transition factor gene exon 14 (METex14) skipping, a recognized oncogenic driver (Bai et al., 2024). Patients with METex14 skipping NSCLC have a shorter survival time compared to other common NSCLC genotypes (Remon et al., 2023). For first-line patients, median progression-free survival (PFS) is 5.0 months under chemotherapy and 3.6 months under immunotherapy, while previously treated patients have median PFS have median PFS of 3.9 and 3.3 months, respectively (Ho et al., 2022).

Recently, METex14 skipping inhibitors have been marketed in China, bringing significant survival benefits. Gumarontinib, a new METex14 skipping inhibitor developed in China, was designated as a breakthrough-therapy-designation (BTD) drug and included in the priority approval channel by the Center for Drug Evaluation (CDE) in September 2021 (Haihe biopharma, 2021). As the only METex14 inhibitor approved for the full-line treatment of METex14 skipping NSCLC, gumarontinib launched in March 2023. The single-arm, multicenter, open-label, phase Ⅱ GLORY study (NCT04270591) demonstrated the overall objective response rate (ORR) of 66%, a median PFS of 8.5 months, and a median overall survival (OS) of 17.3 months (Yu et al., 2023).

Before gumarontinib, savolitinib was the first MET inhibitor approved in China for treating metastatic NSCLC with METex14 skipping alterations in patients who have progressed after or who are unable to tolerate platinum-based chemotherapy (National medical products administration, 2021). A single-arm, open-label, multicenter, Phase II clinical study of salvolitinib (NCT02897479) reported an ORR of 49.2%, a median PFS of 6.8 months, and a median OS of 12.5 months (Lu et al., 2022). Both gumarontinib and savolitinib are recommended by expert consensus as preferred treatment options for METex14 skipping NSCLC (Jun 2023). However, their cost-effectiveness has not been compared. This study aims to evaluate the cost-effectiveness of gumarontinib and savolitinib for treating METex14 skipping NSCLC in China from the perspective of the Chinese healthcare system.

## 2 Methods

### 2.1 Model structure

**FIGURE 1:**
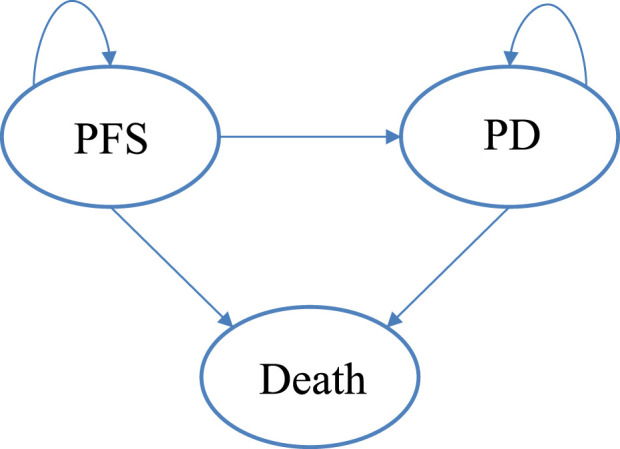
The partition survival model. Abbreviations. PFS, progression-free survival; PD, progression disease.

This study was reported following the *Consolidated Health Economic Evaluation Reporting Standards 2022 guidelines (CHEERS) (* Husereau et al., 2022). A partitioned survival model (PSM) (Figure 1) was established using Microsoft Excel 2019 (https://www.office.com/) to simulate disease progression, aligning with the *National Institute for Health and Care Excellence (NICE) appraisals* (Woods et al., 2017) and *the China Guidelines for Pharmacoeconomic Evaluations 2020* (Liu G and Wu, 2020). PSM is widely used in the economic evaluation of cancer therapy.

The model consisted of three states: progression-free survival (PFS), progressive disease (PD), and death. PFS represented the state in which the patient had not experienced disease progression or death. All patients in this study began the simulation in the PFS state, transitioning to the PD state upon experiencing disease progression. Disease progression was defined according to the Response Evaluation Criteria in Solid Tumors (RECIST) version 1.1 in GLORY study.

Clinical parameters, utility parameters, and cost parameters were included in the model. Clinical parameters comprised the PFS rate, OS rate, and incidence of adverse events (AE) derived through matching-adjusted indirect comparisons (MAIC). Utility parameters included PFS and PD utilities, and disutility of AEs. Cost included direct medical costs, such as drug costs, disease management costs, and AE management costs, as well as end-of-life care for patients in the death state.

The model had a one-month cycle and a lifetime horizon (until 99% of patients died). All costs were based on the 2023 prices and converted into US dollars at the exchange rate of 7.344 yuan per dollar (as of 8 September 2023, 21:00 UTC). Outcomes were discounted at 5% annually, consistent with *the China Guidelines for Pharmacoeconomic Evaluations 2020* (Liu G and Wu, 2020), and a willingness-to-pay (WTP) threshold of $35,007 per QALY gained (three times GDP *per capita* in 2022) was applied.

Key results generated by the model included: (1) the average total cost per patient for gumarontinib and savolitinib; (2) the average life-years (LYs) for patients in each group; (3) the average quality-adjusted life years (QALYs) for patients in each group; (4) the incremental cost-effectiveness ratio (ICER).

### 2.2 Patients population

The trial design, efficacy, and safety details of GLORY and NCT02897479 have been reported in published literature (Lu et al., 2022; Yu et al., 2023). In addition, individual patient data from GLORY were provided by CSPC Pharmaceutical Group Co., Ltd. The target patient population of the model was based on the inclusion criteria of these trials, including (1) adult patients (≥18 years) with histologically or cytologically confirmed locally advanced or metastatic NSCLC (stage IIIb, IIIc, or IV); (2) NSCLC of any histology with METex14 skipping mutations identified by a local or sponsor-designated central laboratory; (3) no EGFR mutation, ALK fusion, ROS1 rearrangement, BRAF mutation, or NTRK fusion; (4) patients who either refused chemotherapy despite being well informed or failed one or two prior lines of systemic therapy for advanced NSCLC; (5) no prior treatment with a MET inhibitor for advanced NSCLC.

Patients in the intervention groups received gumarontinib (300 mg orally daily) until PD, while those in the reference groups received savolitinib (600 mg orally daily) until PD. Since neither the GLORY study nor NCT02897479 studies reported post-progression treatments, this study adopted the standard chemotherapy regimen recommended by *the 2023 Chinese Society of Clinical Oncology (CSCO) guideline for non-small-cell lung cancer* and expert opinions for patients in the PD state. This regimen included pemetrexed (500 mg/m^2^) plus cisplatin (75 mg/m^2^), administrated once every 3 weeks (Oncology Society of Chinese Medical Association, Chinese Medical Association Publishing House, 2022).

### 2.3 Model parameters

#### 2.3.1 Unanchored matching adjusted indirect comparison

Due to the lack of head-to-head clinical research evidence comparing gumarontinib and savolitinib, and because both drugs were conditionally approved based on single-arm trials, conventional indirect comparison methods were not applicable. This study used the unanchored MAIC method to align individual patient data (IPD) of the GLROY with aggregated data (AgD) from NCT02897479 (Signorovitch et al., 2012). The process involved the following steps:

(1) Identify baseline characteristics for matching. Baseline characteristics for cross-trial matching were determined using univariate and multivariate Cox regressions in Stata 17.0, focusing on overall survival (OS) data for gumarontinib. The analysis identified gender and histological subtypes as significant factors (P < 0.05). Additionally, clinical experts and health economists highlighted the importance of patients’ prior systemic treatment history in the real world influencing the OS result. Based on the results of Cox regression and expert opinions, gender, histological subtype, and prior systemic treatment history were selected as the patient’s baseline characteristics for cross-trial matching. Details of the Cox regression analyses are provided in Supplementary Tables S1, S2.
(2) Match baseline characteristics. Propensity score weights were constructed to align the GLORY with the NCT02897479 based on the identified baseline characteristics. Matching results are detailed in Supplementary Table S3.
(3) Compare the efficacy results after matching. After matching, the efficacy and safety data of gumarontinib were recalculated according to the propensity weights, and the survival curves were redrawn. The adjusted and unadjusted efficacy and safety data are presented in Supplementary Tables S4, S5; while the PFS and OS curves are shown in Supplementary Figures S1, S2.

#### 2.3.2 Efficacy

The PFS and OS data of gumarontinib were derived from the individual patient data (IPD) of GLORY after matching adjustment through a MAIC comparison with savolitinib. Survival data for savolitinib were based on simulated IPD reconstructed from published Kaplan-Meier survival curves of NCT02897479 using the Guyot algorithm (Guyot et al., 2012).

The distribution of patients in each health state was obtained from the KM curves of OS and PFS in two trials. The proportion of patients without progression corresponded to the PFS rate, while those with progression were calculated as the difference between the OS and PFS rates. Survival curves beyond the trial observation period were extrapolated using standard parametric models. Several parametric distributions were considered, including exponential, Weibull, Gompertz, log-logistic, and lognormal distributions. The goodness of fit of distributions was evaluated through visual inspection and statistical tests (Akaike information criterion and Bayesian information criterion).

**FIGURE 2:**
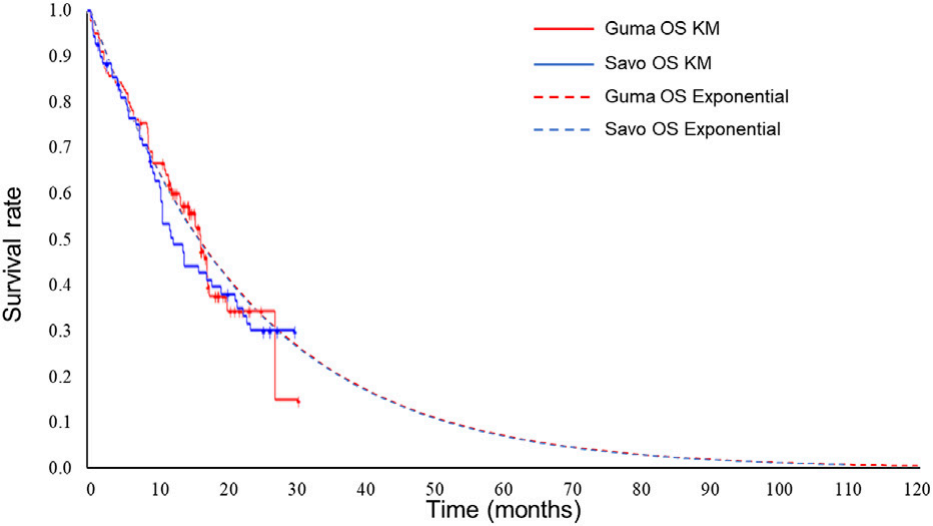
Extrapolated curves and KM curves of OS of gumarontinib and savolitinib (base case analysis). Abbreviations. savo, savolitinib; guma, gumarontinib; KM, Kaplan-Meier.

**FIGURE 3:**
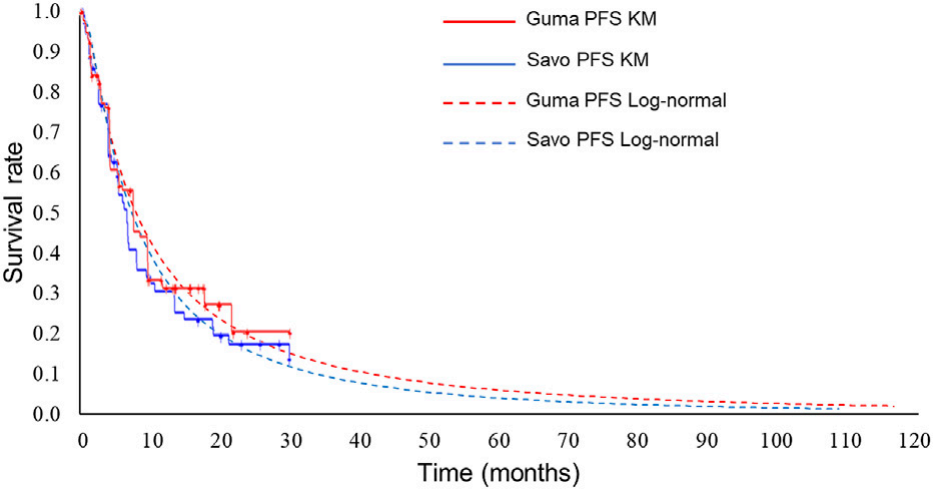
Extrapolated curves and KM curves of PFS of gumarontinib and savolitinib (base case analysis). Abbreviations. savo, savolitinib; guma, gumarontinib; KM, Kaplan-Meier.

Exponential distributions best fit the OS data of both gumarontinib and savolitinib, while the lognormal distributions were optimal for PFS data. The optimal distribution parameters are shown in Table 1, with model fit results shown in Supplementary Tables S6–S9. The extrapolated curves and KM curves of PFS and OS are depicted in Figures 2, 3, and the fitting curves for alternative parametric distributions are illustrated in Supplementary Figures S3–S6.

**TABLE 1:** Optimum fitting distribution and parameters.

Group	Survival curve	Distribution	λ	σ
Gumarontinib	OS	Exponential	0.04397	—
	PFS	Lognormal	2.029	1.321
Savolitinib	OS	Exponential	0.04427	—
	PFS	Lognormal	1.945	1.222

Abbreviations. OS, overall survival; PFS, progression free survival.

#### 2.3.3 Utility

Due to the lack of utility data in both clinical trials, utility values were sourced from an international multicenter study on the health status of stage III/IV NSCLC populations conducted by Nafees et al. (2017). The study showed that the utility values of the Chinese population (n = 76) were 0.804 for “Stable with no side effects” and 0.321 for “Progressive.” Compared to the other countries, the PFS utility in the Chinese population was within the mid-range, while the PD utility was already at its maximum value. Thus, the utility benefit of gumarontinib has not been overestimated. These utility values have been widely cited by Chinese health economists (SU et al., 2023; Sun et al., 2022; Zhang et al., 2022). Also, the disutility of AEs was considered, with most values derived from published literature. For AEs lacking data, hypothetical values were validated by clinical experts, as shown in Table 2.

**TABLE 2:** Base-case model parameter estimates and data sources.

Type of parameter	Parameter	Deterministic (USD)	Low	High	Source
Drug costs (per cycle)	Cycle cost of Gumarontinib	2,921.90	2,377.37	3,352.38	Provided by enterprises
	Cycle cost of savolitinib	3,132.03	2,548.34	3,593.47	Public database^a^
	Cycle cost of Pemetrexed + cisplatin	731.82	595.43	839.63	Public database^a^
Disease management costs (once)	Bed charge	4.15	3.38	4.76	Healthcare price list^b^
	Nursing	4.33	3.52	4.97	Healthcare price list^b^
	Outpatient expenses	2.07	1.68	2.37	Healthcare price list^b^
	Second-generation gene sequencing	496.73	404.16	569.92	Healthcare price list^b^
	CT	21.79	17.73	25.00	Healthcare price list^b^
	Bone scan	54.47	44.32	62.49	Healthcare price list^b^
	Nuclear craniomagnetism	52.12	42.41	59.80	Healthcare price list^b^
	Fibreoptic bronchoscopy	25.65	20.87	29.43	Healthcare price list^b^
	Bronchoscopy	89.19	72.57	102.33	Healthcare price list^b^
	Serological examination	1.58	1.29	1.81	Healthcare price list^b^
	Routine blood test	2.72	2.22	3.12	Healthcare price list^b^
	Urine routine	0.49	0.40	0.56	Healthcare price list^b^
	Routine fecal matter	0.48	0.39	0.55	Healthcare price list^b^
	Electrocardiography	9.34	7.60	10.72	Healthcare price list^b^
AE management costs (once)	Peripheral oedema	0.16	0.13	0.19	Zhang et al. (2023)
	Have a headache	1.94	1.58	2.22	Expert opinion^c^
	Loss of appetite	1.59	1.30	1.83	Expert opinion^c^
	Nauseating	1.59	1.30	1.83	Expert opinion^c^
	Vomiting	1.59	1.30	1.83	Expert opinion^c^
	Elevated alanine aminotransferase	116.29	94.61	133.42	Expert opinion^c^
	Elevated aspartate aminotransferase	116.29	94.61	133.42	Expert opinion^c^
	Have a high-temperature	1.94	1.58	2.22	Expert opinion^c^
	Anemic	29.99	24.40	34.41	Zhang et al. (2023); Ministry of Health, National Development and Reform Commission (2005)
	Hypokalaemia	2.26	1.84	2.59	Expert opinion^c^
	Elevated blood creatinine	21.27	17.31	24.41	Expert opinion^c^
End-of-life care costs (once)	Terminal cost	7,554.01	6,146.25	8,666.94	Zeng et al. (2012)
Utility	PFS	0.804	0.589	0.883	Nafees et al. (2017)
	PD	0.321	0.258	0.366	Nafees et al. (2017)
AE disutility	Oedema	−0.050	−0.041	−0.057	Assumption
	Have a headache	−0.070	−0.057	−0.080	Doyle et al. (2008)
	Loss of appetite	−0.050	−0.041	−0.057	Assumption
	Nauseating	−0.125	−0.101	−0.143	Nafees et al. (2017)
	Vomiting	−0.125	−0.101	−0.143	Nafees et al. (2017)
	Elevated alanine aminotransferase	−0.061	−0.050	−0.070	Sivignon et al. (2020)
	Elevated aspartate aminotransferase	−0.061	−0.050	−0.070	Sivignon et al. (2020)
	Have a high-temperature	−0.416	−0.333	−0.473	Nafees et al. (2017)
	Anemic	−0.119	−0.097	−0.136	Swinburn et al. (2010)
	Hypokalaemia	−0.050	−0.041	−0.057	Assumption
	Elevated blood creatinine	−0.050	−0.041	−0.057	Assumption
Discount	Cost-utility discount rate	0.050	0.041	0.057	Guideline

^a^ Public database: from the China Drug Bidding Database (shuju.menet.com.cn);

^b^ Healthcare price list: from the list of medical service price items of five provinces (average value);

^c^ Expert opinion: from medical oncology departments in 17 Chinese tertiary hospitals.

#### 2.3.4 Cost

From the perspective of the Chinese healthcare system, only direct medical costs were considered, categorized as follows: (1) drug costs; (2) disease management costs; (3) AE management costs; and (4) end-of-life care costs. Both PFS state and PD state contain drug costs, disease management costs, and AE management costs, while the death state included end-of-life care costs in this study, assuming patients received palliative care. Costs were calculated by multiplying the unit costs by the frequencies or days of use per cycle. Based on China’s national medical insurance negotiation requirements, the patient weight and body surface area were assumed to be 60 kg and 1.6 m^2^ respectively in this study. All cost parameters are listed in Table 2.

Drug costs consisted of the costs of gumarontinib, savolitinib, and chemotherapy, which were calculated based on the dosing schedules and the unit costs of the drugs.

Disease management costs encompassed the initial and follow-up visits. Initial visit costs included outpatient service, bed charges, nursing, genetic testing, imaging examination, and bronchoscopy. The genetic testing followed the *Chinese Medical Association guideline for clinical diagnosis and treatment of lung cancer*, recommending 10 genes (EGFR, ALK, ROS1, RET, BRAFV600E, METex14 skipping, MET overexpression, or amplification, HER2, KRAS, and NTRK) for testing (Oncology Society of Chinese Medical Association, Chinese Medical Association Publishing House, 2022). Follow-up visits included serological examination, routine tests (routine blood test, routine urine test, routine stool test), electrocardiograms, and additional bed charges for patients in PD state requiring hospitalization during chemotherapy. The unit prices of each service were derived from healthcare price lists across 5 provinces, and the frequencies were based on expert opinions. Details of disease management costs are shown in Supplementary Table S10.

The costs of AE management were estimated based on AE treatments, durations, and incidences. Only AEs with grade ≥ 3 that required drug management were included. Symptomatic drugs were selected using guidelines (Zhang et al., 2023), expert opinion, and market research data (Supplementary Table S11).

Patients in a terminal state received end-of-life care lasting 1–2 months. Costs were derived from published literature based on the Chinese population (Zeng et al., 2012), with a 5% inflation rate to obtain end-of-life costs in 2023.

### 2.4 Uncertainty analysis

#### 2.4.1 Sensitive analysis

To assess the robustness of the base-case results, deterministic sensitivity analysis (DSA) and probabilistic sensitivity analysis (PSA) were conducted to address the uncertainty in the model.

As for DSA, the discount rate ranged from 0% to 8%, while other parameter estimates varied within their 95% confidence intervals (CI) based on the mean estimate and standard deviation. When the standard deviation was unavailable, it was assumed to be 10% of the mean. DSA results were visualized using tornado diagrams, highlighting the parameters with the greatest influence on model outcomes.

As for PSA, the parametric distribution assumptions followed the recommendations in *Decision Modelling for Health Economic Evaluation* (BRIGGS, 2006). Cost parameters followed a gamma distribution, while AE incidence and utility parameters followed a beta distribution. The shape and scale parameters were calculated using different parameter distribution formulas, as detailed in Supplementary Table S12. Results of PSA were presented via the cost-effectiveness acceptability curve (CEAC), illustrating the probabilities of gumarontinib being cost-effective across various willingness-to-pay (WTP) threshold.

#### 2.4.2 Scenario analysis

Scenario analyses were conducted by dosage adjustment, time horizon, and parametric distributions. The dosage can be adjusted according to the patient’s weight, tolerance and compliance, and other reasons. Since the data of dosage in the real world were challenging to obtain, this study changed the dosage according to the proportion of people with different dosages in the clinical trials (savolitinib: 600 mg: 400 mg = 62: 8; gumarontinib: 300 mg: 200 mg = 84: 8). As for time horizon, a 30-month time horizon scenario analysis was performed using Kaplan-Meier (KM) curves within the gumarontinib trial period without extrapolation (i.e., without any statistical extrapolation). As for parametric distributions, the impact of choosing different parametric distributions for extrapolation was investigated. Specifically, we ran the CEA across all 1,296 possible combinations of six parametric distributions for the OS/ PFS curves across the two MET inhibitors, estimated as: (6 parametric distributions) ^ (2 MET inhibitors × 2 survival curves).

### 2.5 Validation

External and internal validations were conducted to ensure model robustness. For the modeling approach, the use of PSM was aligned with previous CEA for targeting drugs in NSCLC and the use of MAIC was aligned with the previous research method of comparing the efficacy of single-arm targeting drug in the field of METex14 skipping NSCLC. The model structure, assumptions, inputs, and results were validated by clinical experts, pharmacologists, and health economists (Sun et al., 2022; Paik et al., 2022; Carlson et al., 2018). For internal validations, discount rates for costs and efficacy were set to 0% so that undiscounted and discounted LYs, QALYs, and costs were equal. In addition, the results generated by the PSA were compared with the deterministic results to ensure that the mean costs, QALYs, and ICER generated by the PSA were similar to those generated by the model.

## 3 Result

### 3.1 Base case analysis

The health and cost outcomes are shown in Table 3. Compared to savolitinib, gumarontinib provided an incremental gain of 0.10 QALYs (i.e., 1.2 months in perfect health) at an incremental cost of $1,893, resulting in an ICER of $19,343/QALY, which is below the WTP threshold of $35,007/QALY.

**TABLE 3:** Base-case results over a lifetime horizon.

	Guma	Savo	Difference (guma vs. Savo)
Total undiscounted LYs	1.93	1.85	0.08
Total discounted LYs	1.77	1.70	0.07
Total discounted QALYs	1.17	1.07	0.10
PFS	1.00	0.88	0.12
PD	0.17	0.19	−0.02
Total discounted costs	$55,817	$53,925	$1,893
Drug cost	$48,399	$46,458	$1940
PFS	$43,787	$41,137	$2,649
PD	$4,612	$5,321	−$710
Health resource	$599	$613	−$14
AE cost	$0.08	$6.76	−$6.69
End-of-life	$6,820	$6,846	−$26
ICER			$19,343

Abbreviations. LYs, life years; QALYs, quality adjusted life years; PFS, progression-free survival; OS, overall survival; AE, adverse event; ICER, incremental cost-effectiveness ratio.

### 3.2 Sensitivity analysis

**FIGURE 4:**
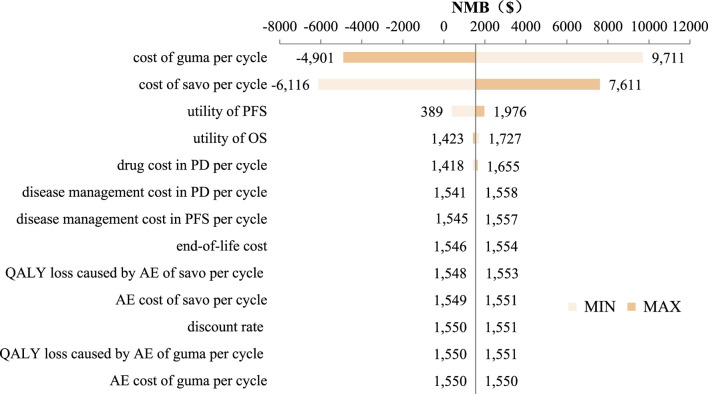
Tornado diagram. Abbreviations. savo, savolitinib; guma, gumarontinib; AE, adverse event; PFS, progression-free survival; PD, progression diease; QALY, quality adjusted life year.

The tornado diagram of DSA is shown in Figure 4. The result showed that the costs of savolitinib and gumarontinib in PFS state and utility values had the greatest impact on the results, while other parameters had minimal influence, confirming the model’s robustness.

**FIGURE 5:**
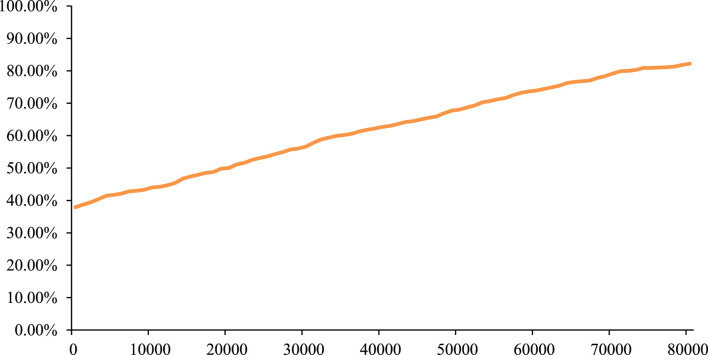
Cost-effectiveness acceptability curve of gumarontinib.

The PSA result showed that, based on 1,000 Monte Carlo simulations, the average incremental QALYs were 0.09, the average incremental cost was $1,922, and the average ICERs was $21,813/QALY, which was similar to the base-case result. In addition, the cost-effectiveness acceptability curve (CEAC) in Figure 5 showed that gumarontinib has a 60.20% probability of being cost-effective under the WTP threshold of $35,007/QALY.

### 3.3 Scenario analysis

After adjusting the dosage of gumarontinib and savolitinib, the drug cost of the gumarontinib group decreased to $52,358, while that of the savolitinib group decreased to $54,548. The incremental cost increased to $2,191, and the ICER increased to $22,273/QALY.

When the time horizon was set to the clinical trial follow-up period instead of the patient’s lifetime, the incremental cost was $417, incremental QALYs were 0.06, and the ICER was $6,868/QALY, as detailed in Table 4.

**TABLE 4:** Scenario analysis results over a lifetime horizon.

	Guma	Savo	Difference (guma vs. Savo)
Total undiscounted LYs	1.46	1.39	0.07
Total discounted LYs	1.39	1.32	0.07
Total discounted QALYs	0.92	0.86	0.06
PFS	0.78	0.72	0.06
OS	0.13	0.14	−0.01
Total discounted costs	$43,095	$43,512	$417
Drug cost	$37,804	$37,356	$448
PFS	$34,195	$33,629	$565
OS	$3,609	$3,724	$-116
Health resource	$468	$458	$10
AE cost	$0.03	$2.32	$-2.30
End-of-life	$5,240	$5,280	$-41
ICER			$6,868

Abbreviations. LYs, life years; QALYs, quality adjusted life years; PFS, progression-free survival; OS, overall survival; AE, adverse event; ICER, incremental cost-effectiveness ratio.

The comprehensive set of analyses across 1,296 possible combinations showed that 51% of ICERs were below the WTP threshold of $35,007, and 72% were below $50,000, indicating that the selection of parameter distribution had a certain impact on the result. However, the model remained relatively robust, as shown in Supplementary Figure S7.

## 4 Discussion

This study evaluated the cost-effectiveness of gumarontinib versus savolitinib in NSCLC patients with METex14 skipping from the perspective of China’s healthcare system. A three-state PSM was constructed, which has been widely used in the economic evaluation of antitumor drugs. Several system reviews showed that the PSM was the most popular model, followed by the markov model, and the decision tree-Markov model (Raad et al., 2024; Wu et al., 2024; Cheng et al., 2024). Clinical data were derived from IPD of the gumarontinib and AgD of the savolitinib via unanchored MAIC. Utility values were derived from published literature, and cost data were obtained from public databases and medical service price catalogs.

Base-case results showed that gumarontinib brought 1.17 QALY at a cost of $55,817 per patient. Compared with savolitinib, the incremental QALYs were 0.10 (i.e., 1.2 months in perfect health), with an incremental cost of $1,893. The PFS efficacy advantage of gumarontinib was the primary contributor to incremental QALYs, while its OS benefit was marginal, and the AE disutilities were negligible. However, the extended PFS period for advantage of gumarontinib led to higher drug costs during PFS ($43,787 vs. $41,137). While gumarontinib incurred lower costs in AE management, medical resources, and end-of-life care, its total costs remained higher than savolitinib.

The study pioneered economic evaluation of MET inhibitors in NSCLC in China and contributed significant insights into gumarontinib and savolitinib’s health and cost outcomes. Innovations include utilizing Cox regression combined with expert opinion and real-world situations to identify baseline characteristics that significantly impact patients’ lifetime outcomes, followed by unanchored MAIC for patient matching. This approach minimized sample loss and ensured stable matching results, especially when sample sizes were small. Although the efficacy of gumarontinib decreased slightly after MAIC, it maintained a lower HR and superior median PFS and OS with savolitinib.

Another feature of this study was the extensive sensitivity analysis. DSA showed that cost and utility values had a significant impact on the results. However, the utility data came from large-scale studies based on the Chinese population, ensuring the robustness of the results to a certain extent. In PSA, this study conducted 1,000 Monte Carlo simulations, demonstrating gumarontinib’s cost-effectiveness probability exceeded 50% at willingness-to-pay (WTP) thresholds above $32,000.

This study is the first economic evaluation of MET inhibitors in Chinese NSCLC patients with METex14 skipping and, therefore, offers a valuable contribution to the emerging understanding of health and cost outcomes associated with gumarontinib and savolitinib in this patient population. At the same time, it can assist China’s medical insurance and health departments in decision-making. A common modeling methodology was applied in the lung cancer field, consistent with the best practices recommended by ISPOR and NICE. The model and all data were carefully reviewed and validated by clinical experts and health economists.

However, some limitations in interpreting the results should be noted. First, an unanchored MAIC was conducted to adjust patient baseline characteristics due to limitations in the design of clinical trials. This method only adjusted for observed differences in patient characteristics but not for unobserved differences in patient characteristics and study design differences. In addition, the unanchored MAIC lacked a common reference drug so that the adjustment results may have certain biases. A future anchored MAIC, leveraging phase III clinical trial data, is recommended.

Second, PSM relies on the extrapolation of survival data to predict patients’ lifetime. Long-term survival data is required to reduce the uncertainty. However, due to the limitations of clinical trials, this study intercepted 30 months of clinical research data, which may lead to a certain bias. The study performed a scenario analysis using data from 30-month clinical trials, which showed incremental costs of $417, incremental QALYs of 0.06, and an ICER of $6,868, which did not affect the conclusions.

Third, this study relied on clinical trials and may fail to demonstrate the real-world situation fully. Real-world studies of savolitinib and gumarontinib showed that some patients may need dosage adjustment due to weight, tolerance, etc. However, in this study, the dosage adjustment was only based on clinical trial proportions, potentially overestimating the drug cost. Accumulating real-world data will allow further exploration of cost and efficacy differences.

Finally, this study did not distinguish between first-line and second-line populations. Due to the small sample sizes, performing MAIC after subgroup analysis would result in sample loss and greater bias. Therefore, this study did not conduct a subgroup analysis, but instead incorporated the subgroup variables into the MAIC process.

## 5 Conclusion

Under the WTP threshold of $35,007/QALY, gumarontinib is more cost-effective compared with savolitinib in treating NSCLC patients with METex14 skipping from the perspective of China’s healthcare system.

## Data Availability

The data analyzed in this study is subject to the following licenses/restrictions: The data that support this study are not publicly available because they contain information that could compromise the privacy of the research participants, but are available from the corresponding author (Wei Li, cpuliwei@cpu.edu.cn) upon reasonable request. Further inquiries can be directed to the corresponding authors. Requests to access these datasets should be directed to Wei Li, cpuliwei@cpu.edu.cn.
