# Supplementary material for: Cost-effectiveness analysis of gumarontinib versus savolitinib for the treatment of advanced or metastatic NSCLC with MET exon 14 skipping mutations in China using partitioned survival model
Source: Front Pharmacol. 2025 Jan 24;16:1400422. doi: 10.3389/fphar.2025.1400422 (PMC11802428; doi:10.3389/fphar.2025.1400422)
Supplement: Supplementary file 1 [file DataSheet1.pdf]

Supplementary Material

1 Supplementary Tables

Supplementary Table 1. Univariate Cox regression for the prognostic value of covariates in GLORY.

**Covariate**	**Specification**	**P value from univariate Cox analysis (OS)**
Age	<75 vs. ⩾75	0.812
Gender	Male vs. female	**0.093**
Smoking history	Smoker vs. never smoker	0.274
ECOG PS	1 vs. 0	0.411
Histology	Adenocarcinoma vs. other	**0.002**
Stage of disease at study entry	III vs. IV	0.353
Brain metastases	Absent vs. present	0.119
Treatment line	1L vs. 2L	0.271

Supplementary Table 2. Multivariate Cox regression for the prognostic value of covariates in GLORY.

**Covariate**	**Specification**	**P value from multivariate Cox analysis (OS)**
Gender	Male vs. female	**0.044**
Histology	Adenocarcinoma vs. other	**0.001**

Supplementary Table 3. Baseline characteristics results before and after MAIC.

Variables	Gumarontinib before MAIC	Gumarontinib after MAIC	Savolitinib
Sample size	92	85	70
Months	58.7%	58.6%	58.6%
Previous treatment, %	46.7%	60.0%	60.0%
Adenocarcinoma	75.0%	57.1%	57.1%

Supplementary Table 4. Efficacy results before and after MAIC.

Endpoints		Gumarontinib before MAIC (95% CI)	Gumarontinib after MAIC (95% CI)	Savolitinib (95% CI)
**PFS**				
	Months	7.7 (7.6 – 11.7)	7.6 (4.3 – 9.7)	6.8 (5.4 – 8.3)
	HR	0.76 (0.51 – 1.11)	0.87 (0.59 – 1.29)	
**OS**				
	Months	17.3 (15.5 – NA)	16.3 (13.3 – NA)	12.4 (10.6 – 21.7)
	HR	0.78 (0.51 – 1.20)	0.93 (0.61 – 1.43)	

Supplementary Table 5. Treatment-related adverse events before and after MAIC.

AEs, n (%)	Gumarontinib before MAIC (95% CI)	Gumarontinib after MAIC (95% CI)	Savolitinib (95% CI)
Oedema	18（20%）	14（16%）	6（9%）
Headache	2（2%）	2（2%）	-
Loss of appetite	1（1%）	2（2%）	-
Nausea	1（1%）	2（2%）	-
Vomit	1（1%）	1（1%）	-
Alanine aminotransferase increased	1（1%）	1（1%）	7（10%）
Aspartate aminotransferase increased	-	-	9（13%）
Fever	-	-	1（1%）
Anemia	-	-	1（1%）
Hypokalemia	-	-	2（3%）
Elevated blood creatinine	-	-	1（1%）

Supplementary Table 6. Statistical testing of gumarontinib OS (after MAIC).

Statistical testing of OS	AIC	BIC
Exponential	**374.730**	**377.252**
Gamma	376.317	381.360

Gompertz	375.255	380.299
Weibull	376.145	381.188
Loglogistic	378.718	383.762
Lognormal	380.926	385.969

Supplementary Table 7. Statistical testing of gumarontinib PFS (after MAIC).

Statistical testing of PFS	AIC	BIC
Exponential	357.557	360.079
Gamma	359.531	364.574
Gompertz	357.455	362.498
Weibull	359.521	364.565
Loglogistic	355.021	360.064
Lognormal	**354.537**	**359.581**

Supplementary Table 8. Statistical testing of savolitinib OS.

Statistical testing of PFS	AIC	BIC
Exponential	**380.812**	**383.061**
Gamma	382.811	387.308
Gompertz	381.711	386.208
Weibull	382.781	387.279

Loglogistic	380.557	385.054
Lognormal	381.211	385.708

Supplementary Table 9. Statistical testing of savolitinib PFS.

Statistical testing of PFS	AIC	BIC
Exponential	364.041	366.290
Gamma	366.041	370.538
Gompertz	358.836	366.402
Weibull	365.760	370.257
Loglogistic	365.760	362.249
Lognormal	**357.752**	**361.877**

Supplementary Table 10. Details of disease management costs.

Items	Frequency/Days	Cost (once)	Source
**First visit**			
Bed	3	4.15	
Nurse	3	4.33	
Outpatient service	1	2.07	
Second-generation gene sequencing	1	496.73	
Contrast enhancement CT	2	21.79	

Bone Scan	1	54.47
Brain MRI	1	52.12
Fiberoptic bronchoscopy	1	25.65
Bronchoscopy	1	89.19
**Return visit (targeting drug)**		
serological examination	1 (every 6 weeks)	1.58
Routine blood test	1 (every 6 weeks)	2.72
Routine urine test	1 (every 6 weeks)	0.49
Routine stool test	1 (every 6 weeks)	0.48
Electrocardiogram	1 (every 6 weeks)	9.34
**Return visit (chemotherapy)**		
Bed	3 (every 3 weeks)	4.15
Outpatient service	1 (every 3 weeks)	2.07
Serological examination	1 (every 3 weeks)	1.58
Routine blood test	1 (every 3 weeks)	2.72
Routine urine test	1 (every 3 weeks)	0.49
Routine stool test	1 (every 3 weeks)	0.48
Electrocardiogram	1 (every 3 weeks)	9.34

Supplementary Table 11. Details of AE management costs.

AE events	Treatment	Cost (once)
Oedema	Furosemide	0.16
Headache	Compound Paracetamol (II)	1.94
Loss of appetite	metoclopramide	1.59
Nausea	metoclopramide	1.59
Vomit	metoclopramide	1.59
Alanine aminotransferase Increased	Magnesium Isoglycyrrhizinate Injection	116.29
Aspartate aminotransferase Increased	Magnesium Isoglycyrrhizinate Injection	116.29
Fever	Compound Paracetamol (II)	1.94
Anemia	red blood cell + Loratadine + Alfacalcidol + vitamin C	29.99
Hypokalemia	Potassium Chloride Injection	2.26
Elevated blood creatinine	HaiKunShenXiJiaoNang	21.27

Supplementary Table 12. Estimation of Scale (α) and Shape (β) parameters.

Distribution	Parameters	formula
Gamma	α	$\frac{\mu^2}{\sigma^2}$
	β	$\frac{\sigma^2}{\mu}$

Beta	α	$\frac{\mu^2(1-\mu)}{\sigma^2} - \mu$
	β	$\alpha \cdot \frac{1-\mu}{\mu}$

2 Supplementary Figures

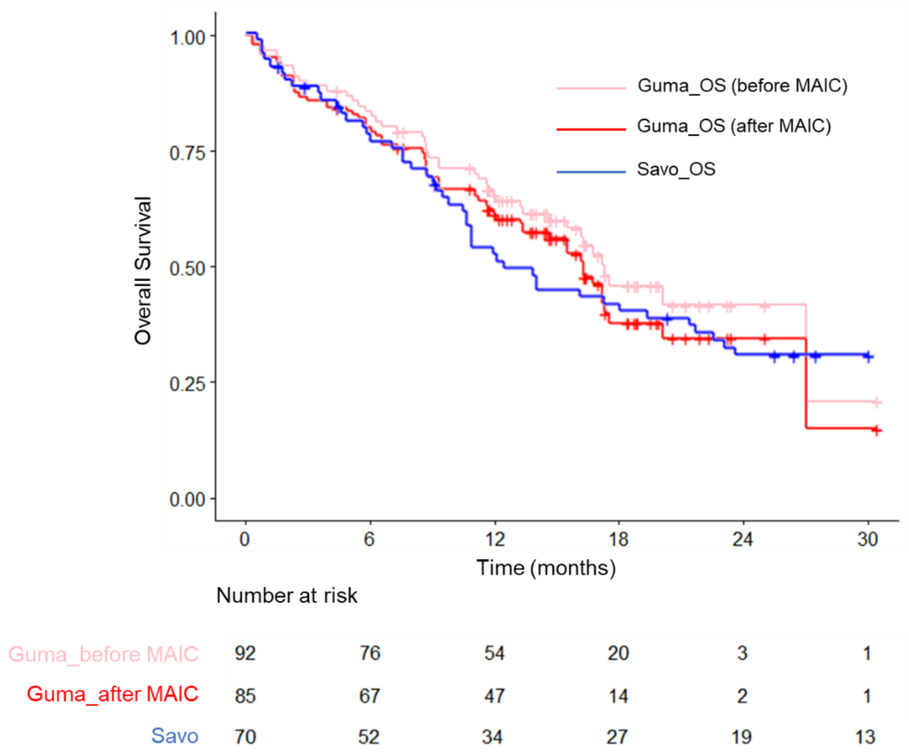

Supplementary Figure 1. Comparison of gumarontinib OS and savolitinib OS

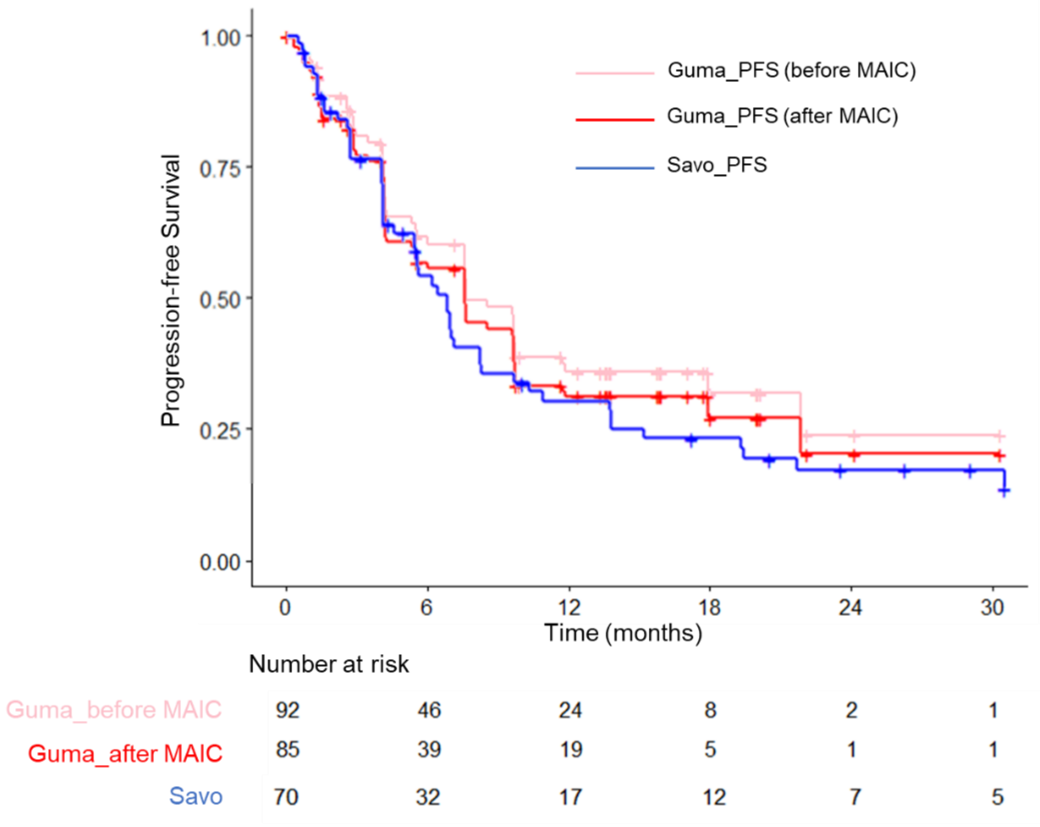

Supplementary Figure 2. Comparison of gumarontinib PFS and savolitinib PFS

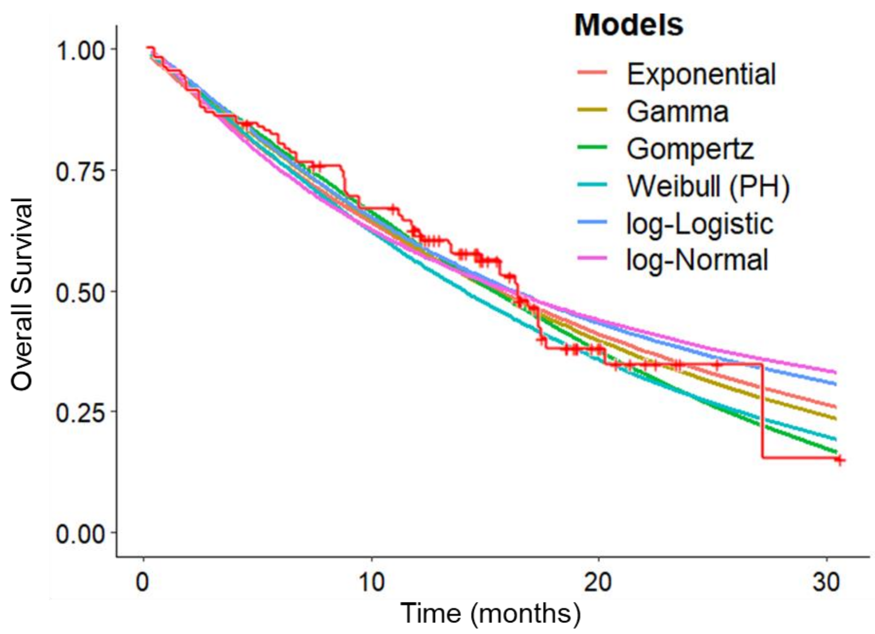

Supplementary Figure 3. Parameter distribution of gumarontinib OS (after MAIC)

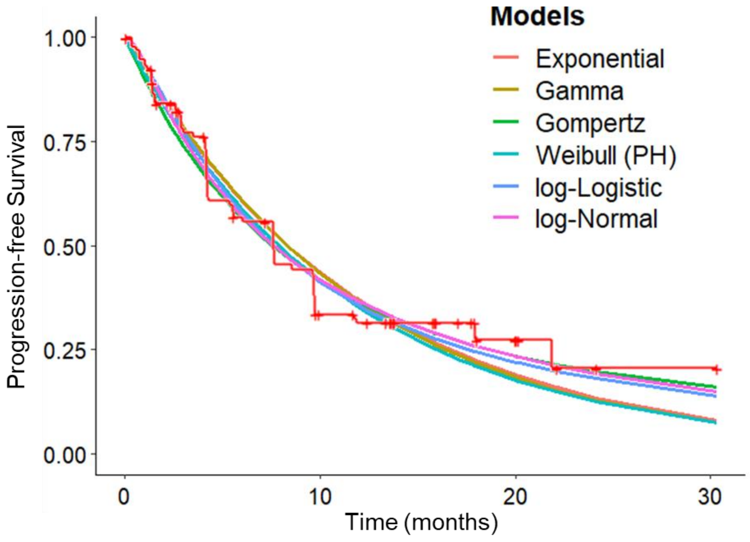

Supplementary Figure 4. Parameter distribution of gumarontinib PFS (after MAIC)

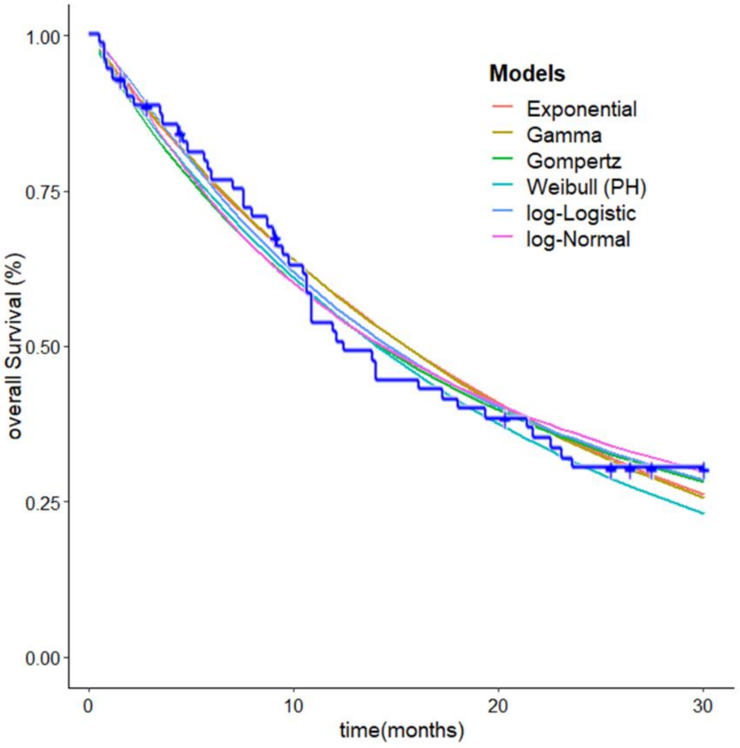

Supplementary Figure 5. Parameter distribution of savolitinib OS

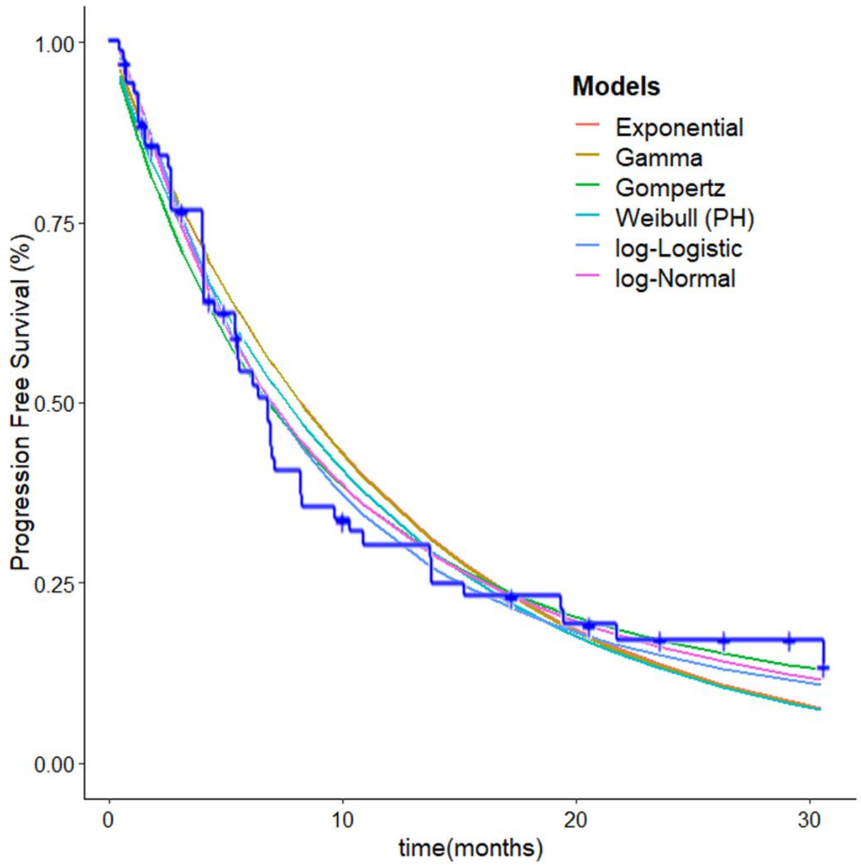

Supplementary Figure 6. Parameter distribution of savolitinib PFS

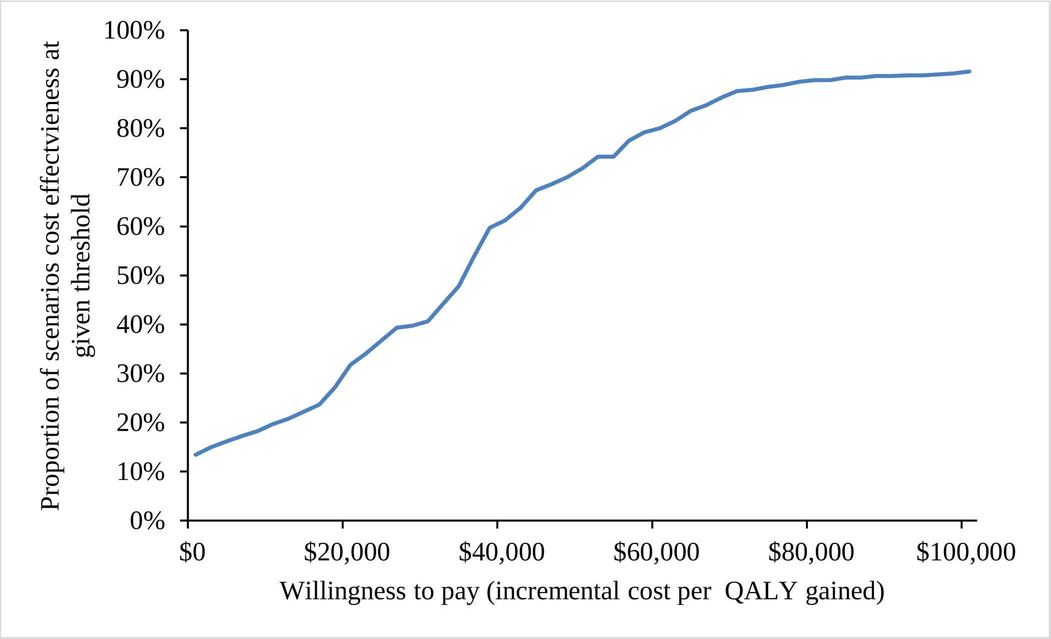

Supplemental Figure 7. Cumulative proportion in which gumarontinib is cost-effective at each willingness-to-pay threshold
